# Older Age Relates to Worsening of Fine Motor Skills: A Population-Based Study of Middle-Aged and Elderly Persons

**DOI:** 10.3389/fnagi.2014.00259

**Published:** 2014-09-25

**Authors:** Yoo Young Hoogendam, Fedde van der Lijn, Meike W. Vernooij, Albert Hofman, Wiro J. Niessen, Aad van der Lugt, M. Arfan Ikram, Jos N. van der Geest

**Affiliations:** ^1^Department of Epidemiology, Erasmus MC University Medical Center, Rotterdam, Netherlands; ^2^Department of Radiology, Erasmus MC University Medical Center, Rotterdam, Netherlands; ^3^Department of Medical Informatics, Erasmus MC University Medical Center, Rotterdam, Netherlands; ^4^Faculty of Applied Sciences, Delft University of Technology, Delft, Netherlands; ^5^Department of Neurology, Erasmus MC University Medical Center, Rotterdam, Netherlands; ^6^Department of Neuroscience, Erasmus MC University Medical Center, Rotterdam, Netherlands

**Keywords:** population-based, fine motor skills, spiral-drawing, elderly, middle-aged, magnetic resonance imaging, cerebellum, cerebrum

## Abstract

**Introduction:** In a population-based study of 1,912 community-dwelling persons of 45 years and older, we investigated the relation between age and fine motor skills using the Archimedes spiral-drawing test. Also, we studied the effect of brain volume on fine motor skills.

**Methods:** Participants were required to trace a template of a spiral on an electronic drawing board. Clinical scores from this test were obtained by visual assessment of the drawings. Quantitative measures were objectively determined from the recorded data of the drawings. As tremor is known to occur increasingly with advancing age, we also rated drawings to assess presence of tremor.

**Results:** We found presence of a tremor in 1.3% of the drawings. In the group without tremor, we found that older age was related to worse fine motor skills. Additionally, participants over the age of 75 showed increasing deviations from the template when drawing the spiral. Larger cerebral volume and smaller white matter lesion volume were related to better spiral-drawing performance, whereas cerebellar volume was not related to spiral-drawing performance.

**Conclusion:** Older age is related to worse fine motor skills, which can be captured by clinical scoring or quantitative measures of the Archimedes spiral-drawing test. Persons with a tremor performed worse on almost all measures of the spiral-drawing test. Furthermore, larger cerebral volume is related to better fine motor skills.

## Introduction

Fine motor skills of the hand are important in many daily activities, such as buttoning a shirt, unlocking doors, or selecting coins from a wallet. If these skills deteriorate, this may give rise to a large variety of minor to major obstacles in daily life. Effects of aging have received much attention for their impact on cognition, either in the context of normal aging or dementia. In particular, aging of the brain quantified by reduced brain volumes or accumulating pathology has been of great interest in explaining cognitive decline (Johnson et al., 2008; Mayda et al., 2011; Bennett et al., 2012). Even though fine motor skills have also been found to decline with aging, population-based studies have been relatively less concerned with describing age effects on fine motor skills in the general population (Smith et al., 1999; Krampe, 2002; Seidler et al., 2010).

Voluntary movements are initiated by cerebral structures, but the cerebellum also plays an important regulatory role in the coordination of movements and balance (Manto, 2008; Shmuelof and Krakauer, 2011). Consequently, patients with selective cerebellar damage present not only with balance problems and uncoordinated movements but also with tremor (Louis et al., 2007; Sullivan et al., 2010a; Bastian, 2011). Thus, both cerebrum and cerebellum are involved in fine motor skills and a coordinated and smooth execution of voluntary movements.

To test fine motor skills the Archimedes spiral-drawing test can be used (Trouillas et al., 1997; Manto, 2010). Conventional assessment of fine motor skills is done using a clinical score, which is based on a visual inspection of the drawing. However, with use of an electronic drawing tablet, automatic quantification of this test can be performed (Pullman, 1998; Miralles et al., 2006; Louis et al., 2012). The spiral-drawing test has been found useful in characterizing movement abnormalities, for example, by quantifying tremor intensity (Haubenberger et al., 2011), or assessing advanced Parkinson disease (Westin et al., 2010), multiple sclerosis (Feys et al., 2009), or Niemann–Pick disease (Hsu et al., 2009).

In the current study, we aimed to describe effects of age on fine motor skills in a general population of middle-aged and elderly persons. For the purpose of this study, we measured fine motor skills using both a qualitative clinical score and quantitative measures obtained from the Archimedes spiral-drawing test. We also wanted to characterize differences of fine motor skills between persons with and without tremor. Furthermore, we aimed to relate cerebellar and cerebral volume to fine motor skills.

## Materials and Methods

### Population

The study was embedded in the Rotterdam study, a prospective, population-based cohort study (*n* = 14,926) that started in 1990 and investigates causes and consequences of age-related disease (Hofman et al., 2011). The institutional review board of Erasmus MC approved the study and participants gave written informed consent. Between January 2009 and December 2010, a random sample of 1,922 persons came to the research center for assessment of fine motor skills using a spiral-drawing test.

### Population for analysis

Of 1,922 persons, we excluded 8 persons based on self-reported Parkinson disease. Self-reported problems of joints in the dominant hand due to osteoarthritis or rheumatoid arthritis were present in 25 persons. Furthermore, in two persons (of which one person is familiar with autosomal dominant cerebellar ataxia) drawings were not suited for our post-processing analyses. Therefore, we chose to exclude these results and 1,912 persons were left for further analyses.

### Fine motor skill assessment

**Figure 1:**
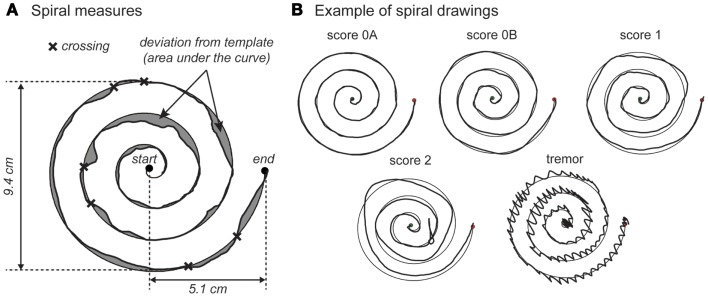
**Examples of spiral-drawing quantification and clinical scores**. **(A)** shows an example of the calculation of quantitative measures of fine motor skills. The start and endpoint are indicated by a dot. The figure explains how deviation from template and crossings are defined. **(B)** shows examples of clinical scores of the spiral drawings with score 0A, 0B, 1, 2, and a tremor.

Fine motor skill was assessed by requiring participants to trace a picture of a spiral template that was printed on a piece of paper attached to an electronic drawing board (WACOM Graphire Wireless Pen Tablet, model CTE-630BT). Participants were instructed to place the pen in the middle of the spiral before the tracing started (Figure 1A). They were not allowed to lean on the drawing board with their hand or arm. Participants were asked to trace the spiral as accurately and as fast as possible using their dominant hand.

### Clinical scoring of spiral drawing

Pen position was recorded at a rate of 60 Hz and stored for offline quantitative analysis. Drawings of participants were evaluated visually to ensure proper data collection. Incomplete drawings were removed from further data processing. Drawings were analyzed visually by a trained observer for qualitative analyses. First, suspected tremors were noted; these drawings were later re-evaluated by an experienced neurologist. Persons with a tremor were included in Table 1 and not included in any further analyses due to the fact that these persons show very different scores on the spiral-drawing variables. Second, each drawing was rated with a clinical score ranging from 0 to 4, according to the Archimedes spiral-drawing test of the international cooperative ataxia rating scale (ICARS) (Trouillas et al., 1997). As this rating scale is intended for patients with movement difficulties, we subdivided score 0: score 0A was given to drawings without any disturbances; score 0B was given to drawings with minor disturbances. Adhering to the ICARS, score 1 is reserved for drawings with impairment and decomposition, score 2 was given to drawings with a line completely drawn outside of the pattern and/or hypermetric swerves. Two persons with a score over 2 were excluded, because quantitative measures could not be reliably obtained from these drawings. Example spiral drawings and associated ratings are shown in Figure 1.

**Table 1:** **Population characteristics**.

	No tremor *n* = 1,888	Tremor present *n* = 24
Age (years)	77.4 ± 6.8	78.1 ± 4.3
Female (%)	60.6%	33.3%*
Primary education only (%)	20.7%	29.2%
Spiral drawing: clinical score		
0A	54.7%	N/A
0B	31.8%	N/A
1	13.0%	N/A
2	0.5%	N/A
Spiral drawing: quantitative data		
Length of drawing (cm)	57.6 ± 3.9	73.9 ± 18.6*
Movement time (s), median (IQR)	16.4 (11.7–22.4)	12.8 (9.8–16.4)*
Average velocity (cm/s), median (IQR)	3.5 (2.6–4.9)	5.8 (4.3–7.6)*
Speed variability, median (IQR)	1.7 (1.3–2.3)	3.8 (3.2–5.2)*
Deviation from template (cm^2^), median (IQR)	5.8 (4.4–7.8)	11.9 (8.4–24.7)*
Number of times crossing template	5.3 ± 2.8	8.0 ± 5.6*
Brain volumes^a^
Intracranial volume (mL)	1462.5 ± 162.7	N/A
Cerebral volume
Cerebral gray matter (mL)	454.8 ± 41.8	N/A
Cerebral white matter (mL)	392.7 ± 52.8	N/A
Cerebellar volume
Cerebellar gray matter (mL)	97.8 ± 10.6	N/A
Cerebellar white matter (mL)	22.0 ± 2.9	N/A
White matter lesion volume (mL)	6.9 (3.5; 14.5)	N/A

*Values are means ± SD or percentages. Median and inter-quartile range (IQR) are given for variables with a skewed distribution*.

*^a^Brain volumes were available in 1,126 persons*.

*Different for tremor and non-tremor when adjusted for age, sex, and level of education (*p* < 0.01).

### Quantitative analysis of spiral drawing

Automatic quantitative analyses were performed using custom-made software written in MatLab (version 8.1; The Mathworks, Natick, MA, USA). This yielded the following outcome measures: movement time (s), defined by the time it took the participant to trace the spiral; length of drawing (cm), defined as the length of the drawn spiral; *average speed*, defined by the ratio of length of drawing and movement time; speed variability (cm/s), defined as the SD of the instantaneous velocity; deviation from template (cm^2^), defined as the area between the template and the drawn spiral; and number of crossings, defined as the number of times the drawn spiral crossed the template (Figure 1A). A smoothly drawn spiral with a clinical score of 0A would have a length of drawing about 56 cm (the length of the template) with little deviation from the template, a low variability in speed, and no crossings (Figure 1B).

### MRI acquisition and image analysis

Magnetic resonance imaging of the brain was performed on a 1.5-T MRI scanner (Signa Excite II, General Electric Healthcare, Milwaukee, WI, USA). The MRI protocol included a high-resolution axial T1-weighted three-dimensional fast radio frequency spoiled gradient recalled acquisition in steady state with an inversion recovery prepulse (FASTSPGR-IR) sequence (TR = 13.8 ms, TE = 2.8 ms, TI = 400 ms, FOV = 25 cm × 25cm, matrix = 416 × 256, flip angle = 20°, NEX = 1, BW = 12.50 kHz, 96 slices with slice thickness 1.6 mm zero-padded to 0.8 mm). All slices were contiguous. According to our standard acquisition protocol images were resampled to 512 × 512 × 192 voxels (voxel size: 0.5 mm × 0.5 mm × 0.8 mm). We also obtained a fluid-attenuated inversion recovery (FLAIR) sequence (TR = 8000 ms, TE = 120 ms, TI = 2,000 ms, FOV = 25 cm × 25 cm, matrix = 320 × 224, NEX = 1, BW = 31.25 kHz, 64 slices with slice thickness 2.5 mm) (Ikram et al., 2011). We used FreeSurfer to obtain total cerebral gray and white matter volumes and total cerebellar gray and white matter volumes (Fischl et al., 2002). White matter lesion volumes were obtained using a *k*-nearest-neighbor classifier, according to a protocol previously described elsewhere (de Boer et al., 2009). Quality control of the brain volumes was performed based on all persons with a completed segmentation. According to a method previously described, outliers were defined as segmentations with an intracranial, cerebral, or cerebellar volume outside a range of ±2.58 SD from the mean, stratified by sex (Hoogendam et al., 2012). A trained observer inspected all outliers (*n* = 214) and a random sample of 500 scans. From the random sample, 9 scans were excluded due to poor segmentation quality due to pathology (e.g., large arachnoid cysts, meningiomas) or technical problems (e.g., motion artifacts, susceptibility artifacts). From the outliers, 31 scans were excluded. The total of 40 persons that were excluded were on average younger (*M* = 66.0, SD = 13.6) than the persons who were included (*M* = 69.0; SD = 10.1); *t* = −1.43, *p* = 0.16. Additionally, in the white matter lesion volume quantifications, five outliers were excluded. Brain volumes were available in 1,126 persons who also performed a fine motor skill assessment.

### Statistical analysis

To test for differences between the persons with or without tremor, we used analysis of covariance with age, sex, and level of education as covariates. Quantitative spiral-drawing measures were standardized using *z*-scores to enable comparison between different variables. Drawing measures with skewed distributions [movement time (s), speed variability of movement (cm/s), deviation from template (cm^2^)] were natural log transformed variables prior to *z*-scoring. To calculate mean scores per 5 years of age when controlling for sex differences, we used analysis of covariance. To establish a linear or quadratic trend, we used regression analyses. We related brain volumes to spiral-drawing measures using regression analyses while adjusting for age, sex, education, and intracranial volume. White matter lesion volumes were natural log transformed because of skewness of the untransformed measure. To aid comparison between volumes, we used *z*-scores of brain volumes. When relating cerebral volume to spiral-drawing measures, we additionally adjusted for cerebellar volume. Conversely, when relating cerebellar volume to spiral drawing, we additionally adjusted for cerebral volume. Leaving out these additional adjustments did not change the associations. We performed additional analyses modeling joint problems as an extra covariate, to test whether this would alter our findings. Analyses were performed using SPSS version 20.0 for Windows. Results are presented with 95% confidence intervals (CI).

## Results

Characteristics of the study population are shown in Table 1. The mean age was 77.4 years with an age range from 48.7 to 96.3 years. In the overall population (*n* = 1,912), 24 persons with had a tremor in the drawing (1.3%). In men (2.1%), tremor occurred more often than in women (0.7%). Furthermore, all electronically collected data of the spiral drawing were different for persons with or without tremor (*p* < 0.01; Table 1). Notably, persons with a tremor had a greater average speed of drawing (median = 5.8 cm/s) compared to persons without tremor (median = 3.5 cm/s).

We correlated spiral-drawing clinical scores and quantitative measures (Table S1 in Supplementary Material). We found that better clinical score showed a small (*r* = 0.06, *p* = 0.02) to moderate correlation (*r* = 0.66, *p* < 0.01) to better quantitative measures of spiral drawing. Since average speed was calculated by dividing length of drawing by movement time, movement time, and average speed were highly correlated, also after correcting for age and sex (*r* = −0.99, *p* < 0.001). Therefore, we did not include average speed in any further analyses.

**Figure 2:**
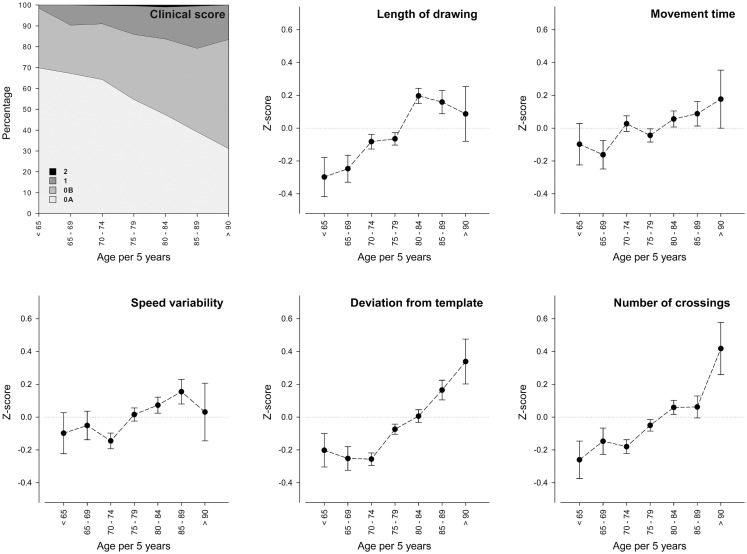
**Age effects on fine motor skills**. The *x*-axis represents age per 5 years and the *y*-axis represents the clinical score or *z*-score of the spiral-drawing measures. Error bars represent SEM. Estimates are adjusted for sex. SD, standard deviation. The number of persons in each age category is *n* = 63 for category <65, *n* = 132 for category 65–69, *n* = 431 for category 70–74, *n* = 628 for category 75–79, *n* = 416 for category 80–84, *n* = 177 for category 85–89, and *n* = 41 for category 90+.

Figure 2 shows spiral-drawing data as function of age, with participants grouped in 5 year intervals. Only nine persons had a clinical score of 2 and this is therefore barely visible in the figure. Overall, with higher age the proportion of persons with a clinical score of 0B, 1, or 2 increased. Regarding the quantitative measures, older age was linearly related to worse performance on all spiral-drawing measures (*p* < 0.001), except for movement time (*p* = 0.07). An additional quadratic effect of age was found for deviation from template (*p* < 0.01). Persons in the age categories over 75 years old started to show an increasing deviation from the template. Adding persons with joint problems of the hand into these analyses, while using joint problems as a covariate, did not alter our results.

In the 1,126 persons without tremor for which MRI scans were available, average intracranial volume was 1462.5 mL (SD = 162.7 mL). Table 2 shows associations between brain volumes and spiral-drawing measures. Larger cerebral gray matter was related to better clinical score (−0.06; 95% confidence interval (CI) −0.010 to −0.02), less speed variability (−0.13; 95%CI −0.24 to −0.01), less deviation from the template (−0.20; 95%CI −0.31 to −0.09), and a smaller number of crossings of the template (−0.12; 95%CI −0.24 to −0.01). Larger cerebral white matter was related to a longer movement time, smaller speed variability, and smaller deviation from the template. Cerebellar gray and white matter volumes were not related to any of the spiral-drawing measures. Larger white matter lesion volume was related to a worse clinical score of the spiral drawing (0.04; 95%CI 0.02–0.07).

**Table 2:** **The association of brain volumes with fine motor skills**.

*n* = 1,126	Clinical score	Length of drawing (cm)	Movement time (s)	Speed variability (cm/s)	Deviation from template (cm^2^)	Number of crossings template
Cerebral gray matter^a^	−**0.06 (**−**0.10;** −**0.02)**	−0.09 (−0.20; 0.02)	0.05 (−0.07; 0.16)	−**0.13 (**−**0.24;** −**0.01)**	−**0.20 (**−**0.31;** −**0.09)**	−**0.12 (**−**0.24;** −**0.01)**
Cerebral white matter^a^	−0.03 (−0.07; 0.01)	0.05 (−0.06; 0.15)	**0.13 (0.02; 0.23)**	−**0.15 (**−**0.26;** −**0.05)**	−**0.14 (**−**0.25;** −**0.04)**	0.02 (−0.08; 0.13)
Cerebellar gray matter^b^	−0.01 (−0.04; 0.02)	0.02 (−0.04; 0.10)	−0.01 (−0.09; 0.07)	0.00 (−0.08; 0.08)	0.03 (−0.05; 0.11)	0.00 (−0.08; 0.08)
Cerebellar white matter^b^	−0.01 (−0.04; 0.01)	0.03 (−0.04; 0.10)	0.04 (−0.04; 0.11)	−0.02 (−0.09; 0.05)	0.01 (−0.06; 0.08)	0.06 (−0.02; 0.13)
White matter lesions	**0.04 (0.02; 0.07)**	0.04 (−0.02; 0.10)	0.00 (−0.07; 0.07)	0.04 (−0.03; 0.10)	0.06 (−0.01; 0.13)	0.01 (−0.05; 0.07)

*Values represent standardized differences in spiral-drawing characteristics per SD increase in brain volume (95% confidence interval). Adjusted for age, sex, education, and intracranial volume*.

*^a^Additionally adjusted for cerebellar volume*.

*^b^Additionally adjusted for cerebral volume*.

*Values in bold indicate significance at level *p* < 0.05*.

## Discussion

Compared to persons without a tremor, persons with a tremor (1.3% of the participants) showed worse performance on most spiral-drawing measures, except that they drew faster in a shorter amount of time. We found that older age was related to a worse performance on all measures of fine motor skill. Furthermore, larger cerebral volume was both related to better clinical score and better quantitative measures of fine motor skills. Larger white matter lesion volume was related to worse clinical score, but not to the quantitative measures. Cerebellar volume was not related to any of our measures of fine motor skills.

Strengths of this study include the population-based design in a large group of middle-aged and elderly and the availability of both clinical scores and electronically obtained spiral-drawing data. Therefore, we were able to give precise estimates of fine motor function in the general elderly population. Furthermore, MRI scans were available in a subsample of the study population, which enabled us to study the relation of brain volumes and fine motor skills in an elderly study sample. Also, note that we used only total cerebral gray and white matter volumes, and no further distinction was made into, e.g., deep and cortical gray matter. An important limitation of the study is the absence of objective diagnoses of Parkinson disease and other diseases of the central or peripheral nervous system that are known to affect motor skills. Unfortunately, we only had data on self-reported Parkinson disease, and one person reported to be diagnosed with autosomal dominant cerebellar ataxia. These persons were removed from the analyses. Adjusting for problems of the joints did not alter our findings. However, we only had data available of self-reported rheumatoid arthritis or osteoarthritis and no other objective measures of problems in the hand or arm that could have influenced performance on the spiral-drawing test.

We found that only 1.3% of persons in a general elderly population had an action tremor. This is low in comparison to most other prevalence studies, although there is a large variation. In a meta-analysis, essential tremor prevalence was estimated to be between 0.4 and 6.3% (Louis and Ferreira, 2010). Establishing reliable tremor prevalence is difficult due to the increasing prevalence with older age and also possible ethnic differences (Louis et al., 2011a). Recently, a community-based study in Brazil reported a prevalence of 17.4% for unspecified tremor in persons over 65 years (Barbosa et al., 2013). Several factors are likely to contribute to our relatively low estimates of tremor occurrence. For instance, our study included younger persons, and participants did not receive a neurological examination. Therefore, we were unable to formally diagnose and distinguish tremor type (Deuschl et al., 1998). Relatively more frequent, a tremor was found in men compared to women. In agreement, sex differences in tremor have been described in adults and children (Louis, 2007; Louis et al., 2011b).

We showed age effects on fine motor skills for both clinical score and quantitative spiral-drawing measures. Since we created standardized scores for the quantitative measures, we were able to compare, which measures were affected more strongly by age. We saw an increase in length of drawing and number of times crossing the template. Movement time and speed variability showed a less steep increase with older age. Linear effects of age were previously found for simpler fine motor tasks such as the Purdue Pegboard and finger tapping tasks (Shimoyama et al., 1990; Ranganathan et al., 2001; Adler et al., 2002). Deviation from the template seemed to stay stable up to age 75, and thereafter showed a more steep increase. Such non-linear effects of age were previously found to affect the amount of time needed to finish demanding fine motor tasks (Smith et al., 1999). Clinical scores detect age effects, but quantitative measures give extra information about movement time and speed variability.

Larger cerebral gray matter was associated with a better clinical score and a lower amount of crossings of the template. Thus, persons with a larger gray matter volume drew with a more stable speed, while persons with a smaller gray matter volume drew with a more varying speed. Larger cerebral white matter volume was related to a longer movement time. Apparently, persons with more cerebral white matter volume took more time to complete the spiral drawing. Furthermore, larger white matter lesion volume was related to worse fine motor skills measured by clinical score, but not to quantitative measures. White matter integrity has been related to fine finger movement (Sullivan et al., 2010b), although other researchers found no relation between white matter hyperintensities and fine motor performance (Gunning-Dixon and Raz, 2000; Fazekas et al., 2005).

Although the cerebellum is known to be involved in fine motor skills, surprisingly neither cerebellar gray matter nor cerebellar white matter volume was related to the spiral-drawing test, which was developed to detect problems in cerebellar functions (Trouillas et al., 1997). Since we had expected to find less variability due to the fact that we used a clinical score in a normal elderly population instead of a patient sample, we had already added an extra category to our clinical score before starting the scoring procedure. Still, this clinical score may not have been sufficiently sensitive to detect an association with cerebellar volume. We were also unable to detect associations between cerebellar volume and quantitative measures of spiral drawing. This leads us to conclude that our measure of cerebellar volume was not sensitive enough to detect any relation to fine motor skills. Particular areas of the cerebellum may show a relation with motor skill as has often been found in functional imaging or lesion studies (Stoodley and Schmahmann, 2009, 2010). In addition, the motor task used in this study may not have been suitable to detect relationships between cerebellar volume and fine motor skills in a general elderly population, especially since it was developed to assess motor skills in patients (Trouillas et al., 1997).

In conclusion, older age was related to worsening of fine motor skills, as was observed by a worsening clinical score. Also, age affected different quantitative measures of the spiral-drawing test that reflect different aspects of fine motor skills. Furthermore, we found associations between larger cerebral volume and better clinical and quantitative spiral-drawing measures. The absence of association between cerebellar volume and fine motor skills is in contrast to common findings of an important cerebellar involvement in fine motor skills. Future studies would benefit from using a more extensive subdivision of cerebellar volume. Relating these subdivisions of the cerebellum to various aspects of fine motor skills may aid provide relevant insights into the etiology of cerebellar malfunction.

## Author Contributions

Substantial contributions to the conception or design of the work (Yoo Young Hoogendam, Meike W. Vernooij, Albert Hofman, Aad van der Lugt, M. Arfan Ikram, and Jos N. van der Geest) or the acquisition (Yoo Young Hoogendam, Fedde van der Lijn, Wiro J. Niessen, and Jos N. van der Geest), analysis (Yoo Young Hoogendam, M. Arfan Ikram, and Jos N. van der Geest), or interpretation of data for the work (Yoo Young Hoogendam, Fedde van der Lijn, M. Arfan Ikram, and Jos N. van der Geest). Drafting the work (Yoo Young Hoogendam, M. Arfan Ikram, and Jos N. van der Geest) or revising it critically for important intellectual content (all authors). Final approval of the version to be published (all authors). Agreement to be accountable for all aspects of the work in ensuring that questions related to the accuracy or integrity of any part of the work are appropriately investigated and resolved (all authors).

## Conflict of Interest Statement

The authors declare that the research was conducted in the absence of any commercial or financial relationships that could be construed as a potential conflict of interest.

## Supplementary Material

The Supplementary Material for this article can be found online at http://www.frontiersin.org/Journal/10.3389/fnagi.2014.00259/abstract
