# Supplementary material for: Older Age Relates to Worsening of Fine Motor Skills: A Population-Based Study of Middle-Aged and Elderly Persons
Source: Front Aging Neurosci. 2014 Sep 25;6:259. doi: 10.3389/fnagi.2014.00259 (PMC4174769; doi:10.3389/fnagi.2014.00259)
Supplement: Supplementary file 1 [file Table1.PDF]

Supplementary table 1. Partial correlations across all spiral drawing measures.
Values represent Pearson r coefficients (p-value) and are adjusted for age and sex.

n = 1,888	Clinical score	Length of the drawing (cm)	Movement time (s)	Average velocity (cm/s)	Speed variability	Deviation from template (cm^2)	Number of times crossings template
Clinical score	1						
Length of the drawing (cm)	0.07 (0.00)	1					
Movement time (s)	-0.45 (0.00)	0.43 (0.00)	1				
Average velocity (cm/s)	0.48 (0.00)	-0.32 (0.00)	-0.99 (0.00)	1			
Speed variability	0.59 (0.00)	-0.16 (0.00)	-0.90 (0.00)	0.92 (0.00)	1		
Deviation from template (cm^2)	0.66 (0.00)	-0.06 (0.01)	-0.54 (0.00)	0.55 (0.00)	0.62 (0.00)	1	
Number of times crossing template	0.10 (0.00)	0.36 (0.00)	0.09 (0.00)	-0.04 (0.06)	-0.03 (0.13)	-0.12 (0.00)	1
